# Adverse perinatal outcomes among practical nurses: The Finnish Medical Birth Register Study

**DOI:** 10.18332/ejm/137355

**Published:** 2021-06-24

**Authors:** Emma Kwegyir-Afful, Reeta Lamminpää, Kimmo Räsänen, Katri Vehviläinen-Julkunen, Tuomas Selander, Mika Gissler

**Affiliations:** 1 School of Health and Society, University of Salford, Manchester, United Kingdom; 2 Department of Nursing Science, Faculty of Health Sciences, University of Eastern Finland, Kuopio, Finland; 3 Institute of Public Health and Clinical Nutrition, Faculty of Health Sciences, University of Eastern Finland, Kuopio, Finland; 4 Science Service Centre, Kuopio University Hospital, Kuopio, Finland; 5 Information Services Department, Finnish Institute for Health and Welfare, Helsinki, Finland; 6 Division of Family Medicine and Primary Care, Department of Neurobiology, Care Sciences and Society, Karolinska Institute, Stockholm, Sweden

**Keywords:** pregnancy, occupational health, birth outcome, practical nurse, nurse assistant

## Abstract

**INTRODUCTION:**

Work as a practical nurse (nurse assistant) may have an effect on pregnancy outcomes. Exposure to chemical, physical and biological hazards are common among hospital personnel. Stressful work conditions such as shift work, prolonged standing and long working hours have been reported among practical nurses. The aim of this study was to examine whether working as a practical nurse is associated with adverse perinatal outcomes.

**METHODS:**

Data were obtained from the Finnish Medical Birth Register of 1997–2014. We included 58512 singleton newborns of practical nurses as cases, and 8765 and 39485 newborns of secretaries and housewives, respectively, as controls. Outcomes included preterm birth (<37 weeks), low birthweight (<2500 g), perinatal death (stillbirth or neonatal death within the first seven days), SGA (<2.5th percentile), and breech presentation, among others. Logistic regression analysis was performed and adjusted for confounders such as maternal age, parity, smoking, and diabetes.

**RESULTS:**

Being a practical nurse had lower likelihood of low birthweight (OR=0.88; 95% CI: 0.81–0.96), perinatal death (OR=0.77; 95% CI: 0.62–0.96), SGA (OR=0.79; 95% CI: 0.72–0.86) and episiotomy (OR=0.90; 95% CI: 0.86–0.94). Practical nursing was significantly related to higher odds of instrumental delivery (OR=1.08; 95% CI: 1.00–1.17), but not with preterm birth, breech presentation, shoulder presentation, or caesarean section.

**CONCLUSIONS:**

After adjusting for confounding variables, working as a practical nurse was associated with higher likelihood of instrumental delivery, particularly vacuum delivery. The risk for shoulder presentation was nearly two-fold compared to controls. Further studies to determine when mothers started their maternity leave and the consequent effect on pregnancy outcome is highly recommended.

## INTRODUCTION

Occupation of the mother has been widely reported as a plausible factor in the causal pathway of adverse birth outcomes^1,2^. Women’s participation in the job market is increasing all the time^3,4^. Practical nurses (nurse assistants) are the seventh most common occupational group in Finland, and 88% are women^5^. In Finland, a total of 105387 women were employed as practical nurses (lähihoitaja in Finnish) in 2016. Their percentage among all working women in Finland in that year was 9.2%^6^. Practical nurses work under the supervision and direction of registered nurses to provide care to the sick^7^. Usually, the work of a practical nurse demands lifting, bathing and feeding patients, among other things. Occupational chemical, physical and biological hazards among hospital personnel has been reported^8-11^. Higher total personal volatile organic compound (VOC) has been reported among practical nurses relative to other health professionals^12^. Besides, other studies have reported negative reproductive consequences as a result of maternal exposure to stressful work demands such as shift work, prolonged standing^13^ and long working hours^14^. However, the association between working as a practical nurse and birth outcomes has not been elucidated nationally or globally. Therefore, in this study we examined whether work as a practical nurse is associated with increased adverse perinatal outcomes.

## METHODS

### Data and source of study cohort

The Finnish Medical Birth Register (MBR) is a national birth register instituted by the National Institute for Health and Welfare (THL) since 1987. It contains information on all births in Finland including both hospital and home deliveries occurring in the country. To ensure its completeness, the MBR data are also linked to the Central Population Register containing all livebirths as well as the Cause-of-Death Register which contains information on stillbirths and infant deaths^15^. The source population included all singleton newborns between 1997 and 2014.

Health outcomes of interest, all taken from the MBR, were preterm birth (<37 weeks), low birthweight (<2500 g), perinatal death (stillbirth or early neonatal death within the first seven days), SGA (<2.5th percentile), breech presentation, caesarean section, vacuum extraction, forceps delivery, episiotomy, and shoulder presentation.

### Determinant of interest

Maternal occupation is routinely collected into the MBR. In Finland, all occupations have been classified by the Statistics Finland’s Classification Services (F-ISCO-88) according to EU directives^16^ and same coding used in the MBR. Work as a practical nurse (F-ISCO 51321) was the determinant of interest. Housewives (coded separately in the MBR) and secretaries (F-ISCO 4115) were the reference categories. We selected housewives and secretaries as controls because their potential exposure to occupationally related biological and chemical factors is less compared to practical nurses. Besides, the work of secretaries is more sedentary compared to that of practical nurses. For the current study cohort, all singleton babies born to practical nurses (n=58512), secretaries (n=8765) and housewives (n=39485) were selected (Table 1).

**Table 1:** Background information of the study population, Finnish Medical Birth Register Data 1997–2014

*Characteristics*	*Practical nurses*		*Secretaries (Reference group 1)*		*Housewives (Reference group 2)*	
	*n*	*%*	*n*	*%*	*n*	*%*
**Total**	58512	54.8	8765	8.2	39485	37.0
**Sex of baby**						
Boy	29816	51.0	4462	50.9	20189	51.1
Girl	28696	49.0	4303	49.1	19296	48.9
**Maternal age**						
<19	81	0.1	3	0.01	469	1.2
19–34	51759	88.5	5988	68.3	31418	79.6
≥35	6672	11.4	2774	31.6	7598	19.2
**Parity**						
Nulliparous	21907	37.5	3358	38.4	2738	6.9
1	18999	32.5	3301	37.7	11077	28.1
2	20611	19.3	1510	17.2	9152	23.2
3	4037	6.9	399	4.6	5793	14.7
4	1541	2.6	101	1.2	3327	8.4
≥5	2048	3.5	86	1.0	7391	18.7
**Marital status**						
Married/registered partnership	30560	52.7	5812	67.1	27711	70.9
Unmarried	25716	44.4	2490	28.8	9875	25.2
Widow	37	0.1	10	0.1	57	0.1
Divorced	904	1.6	177	2.0	963	2.5
Missing	695	1.2	163	1.9	518	1.3
**Smoking**						
No	46000	78.6	7718	88.1	29765	75.4
Quit in first trimester	3536	6.0	181	2.1	1074	2.7
Yes	7556	12.9	651	7.4	7431	18.8
Missing	1420	2.4	215	2.5	1215	3.1

Permission to use the MBR data was granted in 2016–2018 by THL (THL/151/5.05.00/2016/2018) as required by Finland’s data protection legislation.

### Statistical analysis

The data were analysed using IBM SPSS software, version 22. Data were expressed as means with standard deviations or frequencies with percentages. Multivariate adjusted logistic regression analysis was done to compare the prevalence of adverse outcomes between practical nurses relative to housewives and secretaries. To offset the effects of a mother giving birth more than once within the study period, we used generalized estimating equation (GEE) analysis to estimate the adjusted odds ratio (AOR). Based on evidence of potential relationship between birth outcomes and certain factors^17,18^, adjustments were made for covariates such as maternal age, parity, smoking, diabetes and blood pressure. Previous caesarean section was controlled for in the analysis for current caesarean section. Information on these potential confounders were taken from the MBR. Results of these regression analyses are shown as odds ratios (OR) with 95% confidence intervals (CI) (Table 3) with p<0.05 indicating a statistically significant result.

**Table 2:** Prevalence (%) of adverse pregnancy outcomes among newborns of practical nurses, secretaries and housewives, Finnish Medical Birth Register Data 1997–2014 and Finnish Job-Exposure Matrix 1997–2009

*Pregnancy outcome*	*Practical nurses*		*Secretaries (Reference group 1)*		*Housewives ( Reference group 2)*	
	*n*	*%*	*n*	*%*	*n*	*%*
Total	58512	54.8	8765	8.2	39485	37.0
Gestational age (weeks) Mean (SD)	39.77 (1.75)	-	39.75 (1.77)	-	39.71 (1.79)	-
Birthweight (g) Mean (SD)	3.543 (546)	-	3.534 (557)	-	3.577 (565)	-
Preterm birth (<37 weeks)	2687	4.6	425	4.9	1782	4.5
Low birthweight (<2500 g)	1782	3.0	302	3.4	1195	3.0
Perinatal death	207	0.4	38	0.4	213	0.5
SGA (<2.5th percentile)	1765	3.0	287	3.3	1114	2.8
Breech presentation	1504	2.6	291	3.3	722	1.8
Caesarean section	8860	15.1	1653	18.9	4359	11.0
Instrumental delivery (vacuum and forceps deliveries)	3800	6.5	621	7.1	958	2.4
Episiotomy (in vaginal deliveries)	13388	22.9	2491	28.4	4002	10.1
Shoulder presentation	114	0.2	8	0.1	54	0.1

**Table 3:** Comparison of crude and adjusted odds ratios of pregnancy outcomes between newborns of practical nurse (n=58289), secretaries (n=8765) and housewives (n=39485), Finnish Medical Birth Register Data 1997–2014

*Pregnancy outcomes*	*Practical nurses compared to housewives*			*Secretaries compared to practical nurses*		
	*OR (95% CI)*	*AOR (95% CI)*	*GEE analysis AOR (95% CI)*	*OR (95% CI)*	*AOR (95% CI)*	*GEE analysis AOR (95% CI)*
Preterm birth (<37 weeks )	1.02 (0.96–1.08)	0.95 (0.89–1.02)	1.09 (0.97–1.21)	1.08 (0.97–1.20)	0.89* (0.78–1.00)	1.01 (0.94–1.09)
Low birthweight (<2500 g)	1.01 (0.93–1.08)	0.88* (0.81–0.96)	1.18* (1.04–1.34)	1.14* (1.01–1.30)	0.85* (0.73–0.98)	1.07 (0.98–1.17)
Perinatal deaths	0.66* (0.54–0.79)	0.77* (0.62–0.96)	0.79 (0.56–1.12)	0.80 (0.57–1.14)	0.75 (0.51–1.09)	0.82 (0.67–1.01)
SGA (<2.5th percentile)	1.07 (0.99–1.16)	0.79* (0.72–0.86)	0.88 (0.76–1.00)	1.17* (1.02–1.33)	0.74* (0.64–0.86)	0.84 (0.77–0.92)
Breech presentation	1.42* (1.30–1.55)	0.99 (0.89–1.10)	0.78* (0.68–0.88)	1.84* (1.61–2.12)	1.00 (0.85–1.17)	1.06 (0.95–1.17)
Caesarean section	1.44* (1.38–1.50)	1.01 (0.97–1.06)	0.95 (0.89–1.01)	1.87* (1.76–1.99)	0.96* (0.89–1.03)	0.99 (0.94–1.04)
Instrumental delivery (vacuum and forceps deliveries)	2.94* (2.73 – 3.15)	1.08 (1.00–1.17)	1.47* (1.38–1.56)	3.49 (3.07–3.77)	0.87* (0.78–0.98)	1.08* (1.03–1.13)
Episiotomy	2.87* (2.78–2.98)	0.90* (0.86–0.94)	1.47* (1.38–1.56)	4.22* (3.98–4.47)	1.08* (1.00–1.16)	1.081* (1.03–1.13)
Shoulder presentation	0.47* (0.23–0.96)	1.11 (0.78–1.59)	2.01* (0.97–4.14)	0.70* (0.51–0.97)	0.53 (0.24–1.15)	1.13 (0.79–1.61)

AOR: adjusted odds ratio.

* p<0.05.

## RESULTS

The cases were practical nurses (n=58512), with secretaries (n=8765) and housewives (n=39485) as reference groups. The baseline characteristics of the study population are presented in Table 1. There was a higher number of teenage pregnancy (1.2%) among housewives compared to 0.1% among both practical nurses and secretaries. Grand multiparity (five or more pregnancies) was more prevalent (18.7%) among housewives relative to practical nurses (3.5%) and secretaries (1%). Secretaries and housewives were more often married than practical nurses. Smoking during pregnancy was more prevalent (18.8%) among housewives relative to 12.9% among practical nurses and 7.4% among secretaries.

The prevalence of perinatal outcomes is presented in Table 2. The mean gestational age and mean birthweight were similar among all groups. The proportion of preterm births was higher among secretaries (4.9%) and practical nurses (4.6%) than in housewives (4.5%). The proportion of low birthweight was similar among practical nurses and housewives (3.0%) compared to 3.4% among secretaries. The prevalence of perinatal death was relatively similar among all groups. SGA was highest (3.3%) among babies of secretaries followed by practical nurses (3.0%) and housewives (2.8%). The proportion of breech presentation was high among secretaries (3.3%) compared to practical nurses (2.6%) and housewives (1.8%). A higher number of secretaries (18.9%) were delivered of their babies through caesarean section relative to practical nurses (15.1%) and housewives (11.0%). For instrumental delivery, secretaries had the highest proportion of 7.1% compared to 6.5% among practical nurses and 2.4% among housewives. Given episiotomy during vaginal childbirth was relatively higher (28.4%) among secretaries as against 22.9% among practical nurses and 10.1% for housewives.

Table 3 displays the effect estimates from both adjusted odd ratio and GEE models. After adjusting for confounders, risk of preterm birth (OR=0.95; 95% CI: 0.89–1.02) and breech presentation (OR=0.99; 95% CI: 0.89–1.10) were not related to maternal occupation as a practical nurse. The risk of low birthweight (OR=0.88; 95% CI: 0.81–0.96), perinatal death (OR = 0.77; 95% CI: 0.62–0.96), SGA (OR=0.79; 95% CI: 0.72–0.86) and episiotomy in vaginal birth (OR=0.90; 95% CI: 0.86–0.94) were significantly lower among practical nurses compared to housewives. Delivery through caesarean section was similar among practical nurses (OR=1.01; 95% CI: 0.97–1.06) compared to the reference. While being a practical nurse was related to instrumental delivery (OR=1.08; 95% CI: 1.00–1.17) and shoulder presentation (OR=1.11; 95% CI: 0.78–1.59) but the lower limits of the confidence intervals included unity.

Among secretaries, the risk of preterm birth (OR= 0.89; 95% CI: 0.78–1.00), low birthweight (OR=0.85; 95% CI: 0.73–0.98), SGA (OR=0.74; 95% CI: 0.64–0.86), caesarean section (OR=0.96; 95% CI: 0.89–1.03) and instrumental delivery (OR=0.87; 95% CI: 0.78–0.98) were statistically significantly lower compared to practical nurses. But the risk reduction was insignificant for perinatal death (OR=0.75; 95% CI: 0.51–1.09) and shoulder presentation (OR=0.53; 95% CI: 0.24–1.15). The risk of episiotomy during vaginal birth was related to maternal occupation as a secretary (OR=1.09; 95% CI: 1.02–1.17).

## DISCUSSION

In a register-based study of pregnancy outcomes among practical nurses in Finland, we found no increased risk for adverse birth outcomes in practical nurses compared to secretaries and housewives. However, after adjustment for background factors including maternal age, parity, smoking, diabetes, and blood pressure, working as a practical nurse was associated with a higher likelihood of instrumental delivery, particularly vacuum delivery. The risk for shoulder presentation was nearly two-fold compared to controls.

Work in a hospital increases exposure to environmental, biological and chemical risk factors^19^. In a previous study, it was reported that work-related activities including prolonged standing, shift and nightshift work, are associated with adverse pregnancy outcomes^20^. Moreover, in Finland, higher exposure to work-related manual handling of burdens was found to be related to adverse perinatal outcomes^2^. In Denmark, the risk of SGA was found to be higher among jobs with person-lifting^21^. Meanwhile, the work of practical nurses usually entails patient-lifting^22^. In contrast, other studies found no or moderate association between physically demanding work and adverse pregnancy outcomes^23,24^.

In Finland, practical nurses mainly work in shifts and usually the work includes prolonged standing as well as patient lifting. Our results did not identify an association between practical nurses and most adverse pregnancy outcomes compared to the controls. The reasons for the no significant association in the current study may be that mothers were not working during pregnancy and/or there might be protective measures such as lifting devices to prevent person-lifting in hospitals. Besides, in Finland pregnant workers can be reassigned to less demanding duties or they are entitled to paid absence if there is a potential risk to the mother and the foetus^25^.

Additionally, we found a potential risk of instrumental delivery, mainly vacuum, and shoulder presentation deliveries among practical nurses, relative to the controls. The differences regarding these birth outcomes are unexplainable and therefore warrant further investigation.

### Strengths and limitations

Strengths of this study include our large population-based register data from 1997–2014. It has been shown that in Finnish health registries, the validity and coverage are good^26^ and utilization of such data is recommended. Besides, the information of health registries provides a complete and high-quality source of information^27^. We adjusted for confounders based on existing literature. To the best of our knowledge, this is the first study to examine birth outcomes among nurse assistants. Therefore, the results can be applicable globally, especially in settings where there are stringent occupational guidelines protecting pregnant employees similar to Finland.

However, information on whether the mothers were working at the time of the pregnancy or when they went on maternity leave is lacking. Again, due to the secondary source of the data, other factors including work-related biological, psychosocial and chemical stressors that are common among hospital workers^28^ could not be accounted for.

## CONCLUSIONS

This study provides evidence that the risk of most adverse birth outcomes is not elevated among Finnish practical nurses. It is possible that, due to practical nurses’ knowledge about health matters, they may tend to minimise occupational factors that are known to be harmful. Further studies with information on when individual mothers began their maternity leave will be useful.

## Data Availability

The data supporting this research cannot be made available for privacy reasons.

## References

[1] van Beukering MD, van Melick MJ, Mol BW, Frings-Dresen MH, Hulshof CT (2014). Physically demanding work and preterm delivery: a systematic review and meta-analysis. Int Arch Occup Environ Health.

[2] Kwegyir-Afful E, Lamminpää R, Selander T (2018). Manual handling of burdens as a predictor of birth outcome-a Finnish Birth Register Study. Eur J Public Health.

[3] Vanithamani MR, Dinesh Babu N (2019). Impact of Personal Traits and Professional Competencies on Entrepreneurial Competencies of Women Entrepreneurs. Int J Innov Technol Explor Eng.

[4] Abdulwadud OA, Snow ME (2012). Interventions in the workplace to support breastfeeding for women in employment. Cochrane Database Syst Rev.

[5] Appendix table 3c: Most common occupational groups of wage and salary earners aged 18 to 74 without families included in the group of service and sales workers in 2015.

[6] 115q -- Employed persons by occupation group (Classification of Occupations 2010, levels 1 to 5), occupational status, sex and year, 2010-2018.

[7] Saurel-Cubizolles MJ, Kaminski M, Llado-Arkhipoff J (1985). Pregnancy and its outcome among hospital personnel according to occupation and working conditions. J Epidemiol Community Health.

[8] Ahlborg G, Hemminki K (1995). Reproductive effects of chemical exposures in health professions. J Occup Environ Med.

[9] Fransman W, Roeleveld N, Peelen S, de Kort W, Kromhout H, Heederik D (2007). Nurses with dermal exposure to antineoplastic drugs: reproductive outcomes. Epidemiology.

[10] Zhu JL, Knudsen LE, Andersen AM, Hjollund NH, Olsen J (2005). Time to pregnancy among Danish laboratory technicians who were a part of the National Birth Cohort. Scand J Work Environ Health.

[11] Halliday-Bell JA, Quansah R, Gissler M, Jaakkola JJ (2010). Laboratory work and adverse pregnancy outcomes. Occup Med (Lond).

[12] LeBouf RF, Virji MA, Saito R, Henneberger PK, Simcox N, Stefaniak AB (2014). Exposure to volatile organic compounds in healthcare settings. Occup Environ Med.

[13] Teitelman AM, Welch LS, Hellenbrand KG, Bracken MB (1990). EFFECT OF MATERNAL WORK ACTIVITY ON PRETERM BIRTH AND LOW BIRTH WEIGHT. Am J Epidemiol.

[14] Aminian O, Sharifian SAA, Izadi N, Sadeghniiat K, Rashedi A (2014). Association between maternal work activity on birth weight and gestational age. Asian Pacific Journal of Reproduction.

[15] Gissler M, Shelley J (2002). Quality of data on subsequent events in a routine Medical Birth Register. Med Inform Internet Med.

[16] Classification of Occupations 2001.

[17] Lamminpää R, Vehviläinen-Julkunen K, Gissler M, Selander T, Heinonen S (2016). Pregnancy outcomes of overweight and obese women aged 35 years or older - A registry-based study in Finland. Obes Res Clin Pract.

[18] Lamminpää R, Vehviläinen-Julkunen K, Gissler M, Selander T, Heinonen S (2016). Pregnancy outcomes in women aged 35 years or older with gestational diabetes - a registry-based study in Finland. J Matern Fetal Neonatal Med.

[19] Hoskins IA (2003). Environmental and occupational hazards to pregnancy. Prim Care Update Ob Gyns.

[20] Mozurkewich EL, Luke B, Avni M, Wolf FM (2000). WORKING CONDITIONS AND ADVERSE PREGNANCY OUTCOME: A META-ANALYSIS. Obstet Gynecol.

[21] Juhl M, Larsen PS, Andersen PK (2014). Occupational lifting during pregnancy and child's birth size in a large cohort study. Scand J Work Environ Health.

[22] Armstrong BG (1998). Effect of measurement error on epidemiological studies of environmental and occupational exposures. Occup Environ Med.

[23] Aune D, Schlesinger S, Henriksen T, Saugstad OD, Tonstad S (2017). Physical activity and the risk of preterm birth: a systematic review and meta-analysis of epidemiological studies. BJOG.

[24] Pompeii LA, Savitz DA, Evenson KR, Rogers B, McMahon M (2005). Physical exertion at work and the risk of preterm delivery and small-for-gestational-age birth. Obstet Gynecol.

[25] Reijula K, Räsänen K, Hämäläinen M (2003). Work environment and occupational health of Finnish veterinarians. Am J Ind Med.

[26] Gissler M, Haukka J (2004). Finnish health and social welfare registers in epidemiological research. Norsk Epidemiologi.

[27] Gissler M, Surcel HM (2012). Combining health register data and biobank data. Stat J IAOS.

[28] Takeuchi M, Rahman M, Ishiguro A, Nomura K (2014). Long working hours and pregnancy complications: women physicians survey in Japan. BMC Pregnancy Childbirth.

